# Case report: Ectopic production of intact parathyroid hormone (iPTH) by malignoma mimicking primary hyperparathyroidism

**DOI:** 10.3389/fonc.2024.1422131

**Published:** 2024-12-05

**Authors:** Ralph Wendt, Marie Heller, Daniel Härtwig, Sven Oliver Ullmann, Heike Bisanz, Daniela Geister, Luisa Mantovani, Ulrike Hoffmann

**Affiliations:** ^1^ Department of Nephrology, St. Georg Hospital, Leipzig, Germany; ^2^ Department of Pathology, St. Georg Hospital, Leipzig, Germany; ^3^ Department of Oncology and Hematology, St. Georg Hospital, Leipzig, Germany

**Keywords:** IPTH, parathyroid hormone, malignancy, ectopic, primary hyperparathyroidism (pHPT), hypercalcemia

## Abstract

**Background:**

Malignant hypercalcemia is usually caused by osteolytic processes of metastases, production of parathormone-related peptide, or secretion of 1,25-dihydroxyvitamin D. Ectopic PTH (parathyroid hormone) production by malignancy is very unusual.

**Methods:**

Case report and review of the literature.

**Results:**

We present a case of a malignant hypercalcemia with a presentation that mimicked primary hyperparathyroidism in a patient with endometrial carcinoma. Finally, ectopic production of PTH by a rapidly progressive neuroendocrine tumor was proven. Systematic literature review revealed ectopic PTH production by malignancies as an extremely rare cause of hypercalcemia and that most cases were initially misdiagnosed as primary hyperparathyroidism and underwent unnecessary surgical neck exploration in almost all cases.

**Conclusion:**

In patients even with a suggestive constellation of primary hyperparathyroidism, an ectopic paraneoplastic PTH source should be considered if the localization diagnostics are without abnormalities or if the PTH values are unusually high. Concomitant elevated LDH levels should also raise concern about an ectopic malignant source.

## The case

**Figure 1:**
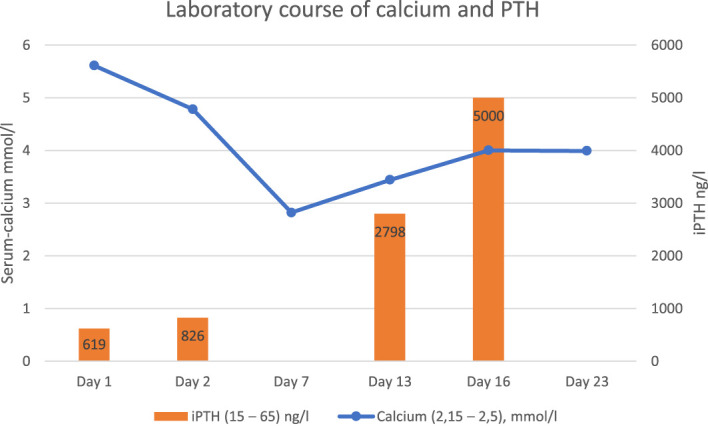
Course of serum calcium and iPTH during hospitalization. Numbers in parentheses represent normal values of laboratory parameters.

**Figure 2:**
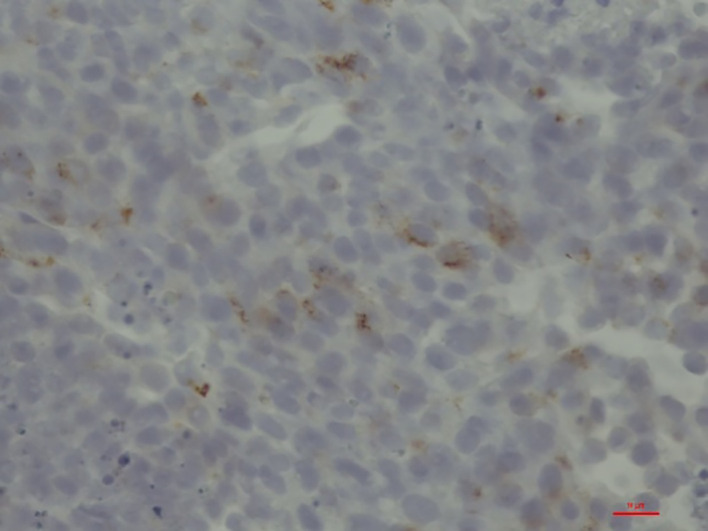
Liver biopsy with large cell, neuroendocrine carcinoma and positive immunostaining for PTH (brown-colored stains).

A 59-year-old female patient presented to the emergency department with weakness, slowness, and impaired consciousness. Routine laboratory investigation showed severe hypercalcemia (5.61 mmol/L) and acute renal failure (complete lab values in **Table 1**). The history of the patient was remarkable of a locally removed endometrial cancer (pT1a pNx R0 L1 V0 Pn0) 4 weeks ago at another hospital. It was described as a localized process which was completely removed. Brachytherapy was already scheduled. Hypercalcemia of malignancy was nevertheless the first differential diagnosis in the emergency department, and the patient was referred to intermediate care and treated with volume expansion, furosemide, and zoledronate. When the surprisingly elevated intact PTH (PTH) values (619 ng/L) were reported, the diagnosis was changed to primary hyperparathyroidism, given the typical constellation and the supposedly cancer-free patient. Parathormone-related peptide (PTHrP) levels were not increased. Parathyroid gland investigations with ultrasound and technetium (99mTc) sestamibi scan did not show an adenoma or any other pathology. A PTH staining from the tissue of the removed endometrial carcinoma showed a negative result. Due to persisting severe hypercalcemia, surgery with exploration of the parathyroid gland was performed on day 13. Histology confirmed normal parathyroid tissue with no evidence of malignancy. After parathyroidectomy, intraoperative intact parathyroid hormone (iPTH) results showed a surprising and massive increase in iPTH levels compared with admission values (iPTH 2,798 ng/L, **Figure 1**). Given those results, an ectopic production of iPTH became obvious. As an incidental finding, a papillary thyroid carcinoma was found in the removed thyroid tissue. Further investigations showed multiple spread of tumor masses, among others also in lung and liver. Liver biopsy revealed a neuroendocrine tumor with histological staining showing a negative result for synaptophysin and chromogranin, but a positive result for PTH (**Figure 2**).

**Table 1:** Laboratory values from admission to day 23.

	Admission	Day 2	Day 7	Day 13	D16	Day 23
Calcium (2.15–2.5), mmol/L	5.61	4.78	2.82	3.44	4.0	3.99
iPTH (15–65) ng/L	619	826		2798	>5000	
Phosphate (0.81–1.45) mmol/L	1.78	1.33	0.51	0.47		0.89
AP (0.58–1.75) µmol/L*s	0.72					12.42
LDH (2.25–3.55), µmol/L*s)	10.2					75.95
Creatinine (<88), µmol/L	109	147	106	52		65
eGFR (CKD-EPI), mL/min/1.73 m²	48	33	49	111		86

AP, alkaline phosphatase in serum. Numbers in parentheses represent normal values of laboratory parameters.

Despite repeated bisphosphonate therapy and chemotherapy, the patient died of her serious illness within a short time (day 36 of hospitalization).

## Discussion and review of the literature

Hypercalcemia of malignancy is usually caused by osteolytic processes of metastases, production of parathormone-related peptide, or secretion of 1,25-dihydroxyvitamin D. Here, we present a case of ectopic PTH production by malignancy and have identified further 11 published cases in the literature.

Cancer-associated hypercalcemia has been classified into four subtypes: humoral, local osteolytic, 1,25-dihydroxyvitamin D-mediated, and ectopic hyperparathyroidism (1).

Humoral hypercalcemia of malignancy is typically caused by tumor secretion of parathyroid hormone-related protein (PTHrP). This increases osteoclastic bone resorption and renal tubular reabsorption of calcium by binding the parathyroid hormone (PTH)-PTHrP type 1 receptor in the bones and kidneys.

Local osteolytic hypercalcemia, which is characterized by extensive bone metastasis, is due to tumor cells in bone producing cytokines that increase osteoclastic bone resorption and suppress osteoblastic bone formation.

In ectopic hyperparathyroidism, tumors produce parathyroid hormone (PTH), which can act alone or in concert with PTHrP to stimulate bone resorption (5–15).

With hypercalcemia mediated by excess production of 1,25-dihydroxyvitamin D, tumors upregulate the expression 1-alpha-hydroxylase, the enzyme that converts 25-hydroxyvitamin D to 1,25-dihydroxyvitamin D. Excess 1,25-dihydroxyvitamin D increases intestinal calcium absorption and bone resorption.

Primary hyperparathyroidism (pHPT) is the most frequent cause for hypercalcemia in the general population. Even in patients with cancer who had hypercalcemia, 6% to 21% had concomitant primary hyperparathyroidism (2, 3). In 675 consecutive patients with a biochemical diagnosis of PHPT who underwent surgical parathyroidectomy, the majority of patients had preoperative PTH levels of 100 pg/mL–400 pg/mL (433 patients), but 55 patients (8.1%) had PTH levels >400 pg/mL with maximum values up to 1,521 pg/mL (4). Therefore, the high values of iPTH in our case (619 ng/L) together with severe hypercalcemia was suggestive of pHPT and not very unusual. As depicted in **Table 2**, PTH levels in the published cases with ectopic PTH production were in at least 50% of the cases lower than 10× the ULN. The normal serum phosphate was confounded by acute renal failure. After improvement and normalization of renal function, serum phosphate levels dropped tremendously as expected with high iPTH levels. Remarkedly, the elevated value for lactate dehydrogenase (LDH 10.2 µmol/L*s (2.25–3.55)) already at admission should have raised suspicion for another cause of hypercalcemia other than pHPT.

**Table 2:** Summary of all published cases with ectopic PTH production in malignancies with addition of our present case.

Author, year	Malignancy	Max PTH	Max. calcium mmol/L	LDH	Surgical exploration of the neck	Outcome
Yoshimoto 1989 (5)	Small cell lung carcinoma	4,650 ng/L (230–630)	4.84	NR	Yes (postmortem)	Deceased day 2
Wong 2005 (6)	Rhabdomyosarcoma	62.22 pmol/L (0.74–5.62)	3.89	NR	No	Deceased within 2 months
Van Houten 2006 (7)	Neuroendocrine carcinoma of the pancreas	2310 pg/mL (6-40)	4.49	NR	Yes	Deceased within a few days
Strewler 1993 (8)	Neuroectodermal malignancy	290 ng/L (15–65)	3.72	NR	Yes	Not reported
Rizzoli 1994 (9)	Thymoma	8.9 pmol/L (1.0–6.0)	2.9	NR	Yes	Not reported
Nussbaum 1990 (10)	Ovarian cancer	429 ng/L (10–60)	3.9	NR	Yes	Survived > 4 months
Nielsen 1996 (11)	Squamous cell lung carcinoma	560 ng/L (10–50)	2.5 (ion. Ca). 1.15–1.35)	NR	Yes	Deceased in 3 weeks
Iguchi 1998 (12)	Papillary thyroid adenocarcinoma	9,800 pg/mL (160–520)	2.87	206 IU/L (200–370)	Yes	Survived > 3.5 years
Goldman 1978 (13)	Lymphoma	2.8 mµg/mL (1.0–2.0)	3.69	280 U/ml (85–225)	Yes	Deceased day 7
Eid 2004 (14)	Transitional cell carcinoma of the urinary bladder	432 pg/mL (13-65)	2.99	NR	Yes	Deceased within a few weeks
Neves 2023 (15)	Small cell lung carcinoma	117 pg/mL (10–65)	4.02	705 U/L (135–225)	No	Not reported
Wendt 2024	Neuroendocrine tumor	5,000 ng/L (15–65)	5.61	10.2 µmol/L*s (2.25–3.55)	Yes	Deceased d36

Numbers in parentheses represent normal values of laboratory parameters.

## Conclusion

In conclusion of our case and after the extensive review of all published cases of ectopic PTH production by malignancies, we propose the following:

1. In cases of high iPTH (>400 pg/mL or ng/L) with suspicion of PHPT, but no signs of pathology of the parathyroid gland on ultrasound or parathyroid technetium (99mTc) sestamibi scan, we advise to undergo an extensive screening for malignancies before neck exploration.
2. In cases with suspicion of PHPT, but no signs of pathology of the parathyroid gland on ultrasound or parotid technetium (99mTc) sestamibi scan, and an elevated LDH (>3× ULN) and no other obvious reason for LDH elevation, do an extensive screening for malignancies before neck exploration.

## Data Availability

The original contributions presented in the study are included in the article. Further inquiries can be directed to the corresponding author.

## References

[1] Guise TA Wysolmerski JJ . Cancer-associated hypercalcemia. N Engl J Med. (2022) 386:1443–51. doi: 10.1056/nejmcp2113128 35417639

[2] Strodel WE Thompson NW Eckhauser FE Knol JA . Malignancy and concomitant primary hyperparathyroidism. J Surg Oncol. (1988) 37:10–2. doi: 10.1002/jso.2930370104 3336214

[3] Hutchesson ACJ Bundred NJ Ratcliffe WA . Survival in hypercalcaemic patients with cancer and co-existing primary hyperparathyroidism. Postgrad Méd J. (1995) 71:28–31. doi: 10.1136/pgmj.71.831.28 7708588 PMC2397910

[4] Hargitai L Bereuter CM Dunkler D Geroldinger A Scheuba C Niederle B . The value of intraoperative parathyroid hormone monitoring in patients with primary hyperparathyroidism and varying baseline parathyroid hormone levels. BJS Open. (2022) 6:zrac118. doi: 10.1093/bjsopen/zrac118 36515670 PMC9749480

[5] Yoshimoto K Yamasaki R Sakai H Tezuka U Takahashi M Iizuka M . Ectopic production of parathyroid hormone by small cell lung cancer in a patient with hypercalcemia. J Clin Endocrinol Metab. (1989) 68:976–81. 10.1210/jcem-68-5-976 2541161

[6] Wong K Tsuda S Mukai R Sumida K Arakaki R . Parathyroid hormone expression in a patient with metastatic nasopharyngeal rhabdomyosarcoma and hypercalcemia. Endocrine. (2005) 27:83–6. doi: 10.1385/endo:27:1:083 16077176

[7] VanHouten JN Yu N Rimm D Dotto J Arnold A Wysolmerski JJ . Hypercalcemia of Malignancy due to ectopic transactivation of the parathyroid hormone gene. J Clin Endocrinol Metab. (2006) 91:580–3. doi: 10.1210/jc.2005-2095 16263810

[8] Strewler GJ Budayr AA Clark OH Nissenson RA . Production of parathyroid hormone by a Malignant nonparathyroid tumor in a hypercalcemic patient. J Clin Endocrinol Metab. (1993) 76:1373–5. doi: 10.1210/jcem.76.5.7684395 7684395

[9] Rizzoli R Pache JC Didierjean L Bürger A Bonjour JP . A thymoma as a cause of true ectopic hyperparathyroidism. J Clin Endocrinol Metab. (1994) 79:912–5. doi: 10.1210/jcem.79.3.8077382 8077382

[10] Nussbaum SR Gaz RD Arnold A . Hypercalcemia and ectopic secretion of parathyroid hormone by an ovarian carcinoma with rearrangement of the gene for parathyroid hormone. N Engl J Med. (1990) 323:1324–8. doi: 10.1056/nejm199011083231907 2215618

[11] Nielsen PK Rasmussen AK Feldt-Rasmussen U Brandt M Christensen L Olgaard K . Ectopic production of intact parathyroid hormone by a squamous cell lung carcinoma. Vivo vitro. J Clin Endocrinol Metab. (1996) 81:3793–6. doi: 10.1210/jcem.81.10.8855839 8855839

[12] Iguchi H Miyagi C Tomita K Kawauchi S Nozuka Y Tsuneyoshi M . Hypercalcemia caused by ectopic production of parathyroid hormone in a patient with papillary adenocarcinoma of the thyroid gland. J Clin Endocrinol Metab. (1998) 83:2653–7. doi: 10.1210/jcem.83.8.5025 9709927

[13] Goldman JW Becker FO . Ectopic parathyroid hormone syndrome. Occurrence in a case undifferentiated lymphoma with bone marrow involvement. Arch Intern Med. (1978) 138:1290–1. doi: 10.1001/archinte.138.8.1290 677991

[14] Eid W Wheeler TM Sharma MD . Recurrent hypercalcemia due to ectopic production of parathyroid hormone-related protein and intact parathyroid hormone in A single patient with multiple Malignancies. Endocr Pr. (2004) 10:125–8. doi: 10.4158/ep.10.2.125 15256329

[15] Neves A Mendonça I Marques JAC Costa J Almeida JS . Malignant hypercalcemia induced by the ectopic production of intact parathyroid hormone (PTH). Cureus. (2023) 15:e34770. doi: 10.7759/cureus.34770 36909108 PMC10001420

